# Acute subdural haematoma exacerbates cerebral blood flow disorder and promotes the development of intraoperative brain bulge in patients with severe traumatic brain injury

**DOI:** 10.1186/s40001-023-01100-y

**Published:** 2023-03-27

**Authors:** Shangming Zhang, Qizuan Chen, Liang Xian, Yehuang Chen, Liangfeng Wei, Shousen Wang

**Affiliations:** 1 Fuzong Clinical Medical College of Fujian Medical University, Fuzhou, 350025 China; 2 Department of Neurosurgery, 900TH Hospital of Joint Logistics Support Force, Fuzhou, 350025 China

**Keywords:** Decompressive craniectomy, Diffuse brain swelling, Intracranial pressure, Intraoperative brain bulge, Subdural haematoma, Traumatic brain injury

## Abstract

**Background:**

Decompressive craniectomy (DC) is a routine procedure used for the treatment of severe traumatic brain injury (TBI) with concomitant acute subdural haematoma (SDH). However, certain patients are prone to developing malignant brain bulge during DC, which prolongs the operative time and worsens patient outcomes. Previous studies have shown that malignant intraoperative brain bulge (IOBB) may be associated with excessive arterial hyperaemia caused by cerebrovascular system disorders. Through a clinical retrospective analysis and prospective observations, we found that the cerebral blood flow of patients who possessed risk factors manifested high resistance and low flow velocity, which severely affected brain tissue perfusion and resulted in the occurrence of malignant IOBB. In the current literature, rat models of severe brain injury-associated brain bulge have rarely been reported.

**Methods:**

To gain an in-depth understanding of cerebrovascular changes and the cascade of responses related to brain bulge, we introduced acute SDH into the Marmarou model for the preparation of a rat model of high intracranial pressure (ICP) to simulate the pathological conditions experienced by patients with severe brain injury.

**Results:**

With the introduction of a 400-µL haematoma, significant dynamic changes occurred in ICP, mean arterial pressure, and relative blood perfusion rate of the cerebral cortical vessels. ICP increased to 56.9 ± 2.3 mmHg, mean arterial pressure showed reactive decrease, and the blood flow of cerebral cortical arteries and veins on the non-SDH-affected side decreased to < 10%. These changes could not fully recover even after DC. This resulted in generalised damage to the neurovascular unit and a lag effect to the venous blood reflux, which triggered malignant IOBB formation during DC.

**Conclusion:**

An excessive increase in ICP causes cerebrovascular dysfunction and brings about a cascade of damage to brain tissue, which forms the basis for the development of diffuse brain swelling. The subsequent heterogeneous responses of the cerebral arteries and veins during craniotomy may be the main cause of primary IOBB. Clinicians should pay particular attention to the redistribution of CBF to various vessels when performing DC in patients with severe TBI.

## Background

In patients with severe brain injury, secondary injury such as the space-occupying effect of intracranial haematomas, inflammation, and a cascade of damage to cells may occur within minutes after the primary trauma-induced injury, ultimately manifesting as a continuous increase in intracranial pressure (ICP) that urgently necessitates decompressive craniectomy (DC) [1, 2]. However, in a portion of patients, especially those with concomitant acute subdural haematoma (SDH), surgeons often observe varying extents of brain bulge towards the outside of the bone window within a short period of time after haematoma evacuation via a bone flap, which severely curtails the outcome level of patients [3, 4]. There are many causes of brain bulge. In cases where the occurrence of contralateral haematoma after craniotomy results in the protrusion of brain tissue outside the bone window, the cause of brain bulge is exogenous or secondary and may be remediated by secondary surgery. However, in certain patients, bulging of brain tissue occurs rapidly despite the adoption of a series of ICP-reducing measures. The cause of such cases may be primary or originate from within the brain tissue rather than the compression effect of recurrent haemorrhage. Therefore, adequate preoperative prediction of patients who may develop malignant brain bulge and an in-depth understanding of this phenomenon are highly necessary for surgeons. However, most clinical studies on malignant intraoperative brain bulge (IOBB) during DC have been focused on risk factors and handling measures. Although many researchers have attempted to elucidate the relevant pathophysiological mechanisms [5–7], there remains a lack of clear theories capable of explaining all the characteristics of this phenomenon.

In this study, we systematically analysed the high-risk factors of primary brain bulge in patients with traumatic brain injury (TBI) and monitored changes in middle cerebral artery blood flow to provide a reference for surgeons. Based on the revelations provided by the clinical cases, we subsequently aimed to investigate the possible mechanisms of primary brain bulge. A rat model of diffuse brain swelling (DBS) was established by combining two types of rat models of disease to simulate the actual conditions faced by clinical patients, and the IOBB phenomenon and cerebrovascular responses during DC were evaluated.

## Methods

### Clinical retrospective data

The data of patients with severe TBI who were admitted to the 900th Hospital between January 2017 and January 2022 were retrieved. The inclusion criteria were as follows: severe TBI with concomitant acute SDH, surgical indications for craniotomy for haematoma evacuation and ICP reduction, age ≥ 16 years, and written informed consent provided personally or by family members. The exclusion criteria were as follows: concomitant severe multiple chest and abdominal injuries, shock, history of multi-organ insufficiency, long-term anticoagulant or antiplatelet therapy, definite intracranial tumour and vascular disease, and patients with definite recurrent contralateral intracranial haemorrhage, i.e. those with exogeneous or secondary brain bulge. Malignant IOBB was defined as the gradual protrusion of brain tissue outside the bone window margin shortly after intraoperative dura mater opening, with the brain tissue noticeably trapped and compressed by the bone window margin and unable to be restored, and the adoption of measures such as hyperventilation and (or) dehydrating agents being ineffective in controlling bulging. Data acquired for the study were as follows: dependent variable (presence or absence of IOBB) and independent variables (sex, age, preoperative Glasgow Coma Scale [GCS] score, presence or absence of concomitant multiple contusions and lacerations in bilateral brain tissue, presence or absence of concomitant subarachnoid haemorrhage (SAH) or intraventricular haemorrhage, presence or absence of bilateral basal cistern compression and disappearance, SDH thickness, and ICP values before craniotomy).

### Cerebral blood flow (CBF) monitoring of clinical patients

**Fig. 1:**
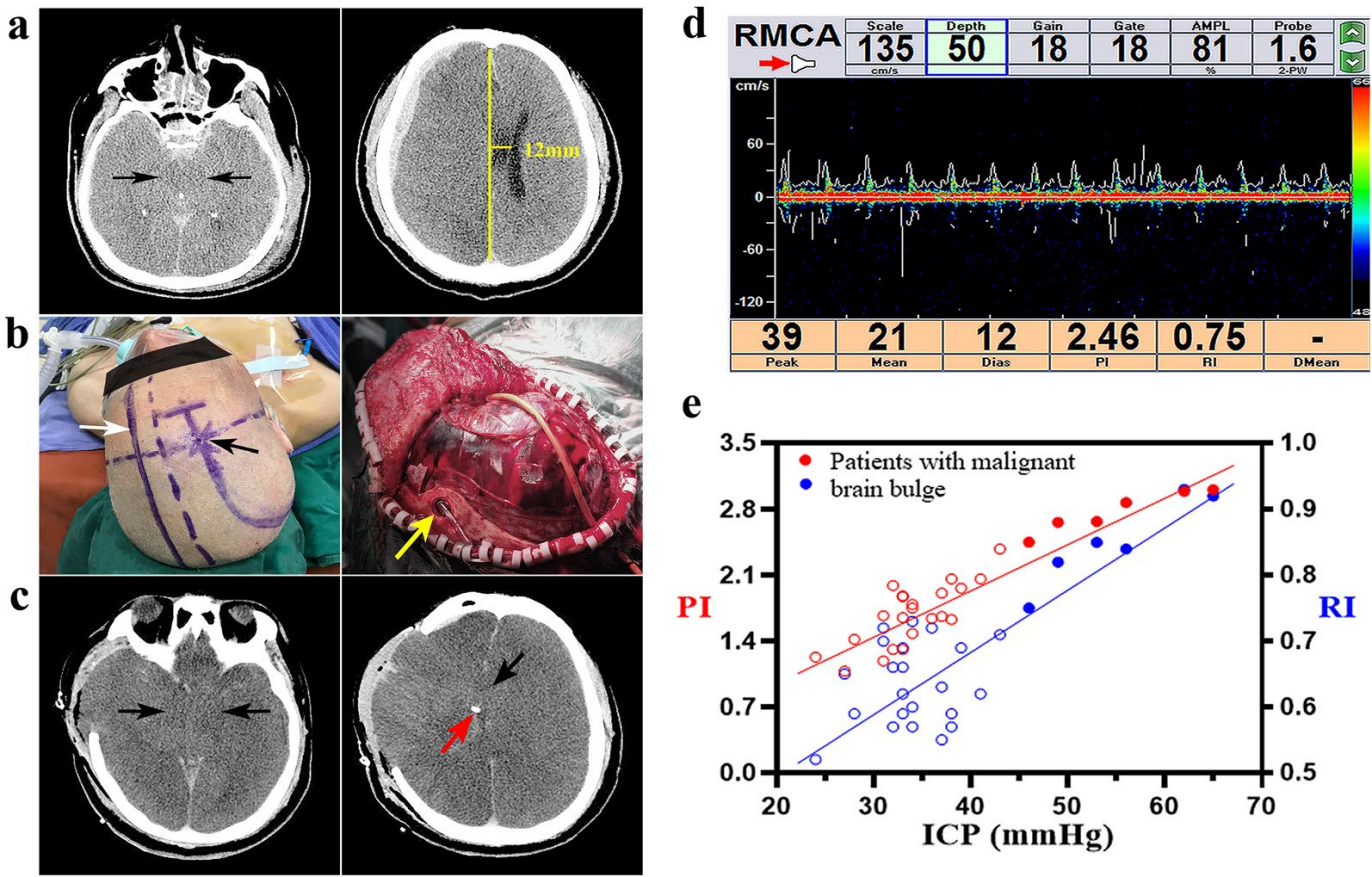
Monitoring of middle cerebral artery blood flows of 28 patients included in the study. **a** Preoperative CT images of a 40-year male patient shows the presence of SDH in the right brain, disappearance of bilateral basal cisterns (black arrows) and a midline shift of 12 mm (yellow line). **b** When standard DC was performed, the skin was first incised at Kocher’s point (black arrow). A cranial burr hole was then created and puncturing was performed in the direction of the given sagittal plane (white arrow) to puncture the frontal horn of the ventricular system for the acquisition of ICP data. In this case, the measured ICP was 46.2 mmHg. Severe brain bulge occurred 15 min after the intraoperative cutting of the dura mater, with brain tissue protruding more than 4 cm beyond the bone window. The yellow arrow indicates the cranial hole used for the implantation of the ICP probe. **c** After emergency closure of the cranial cavity, a CT re-examination revealed the presence of brain bulge beyond the bone window, complete disappearance of the basal cistern and ventricle (black arrows), and accurate positioning of the ICP probe in the ventricle (red arrow). **d** Before making the scalp incision, the middle cerebral artery blood flow was monitored through the temporal window, and the values of relevant haemodynamic parameters were recorded. **e** Scatter plot and correlation analysis of preoperative ICP and PI/RI values of middle cerebral artery flow on the SDH side of the 28 TBI patients. PI = 0.05035 × ICP − 0.02721, *r* = 0.9316, *P* < 0.0001, *n* = 28. RI = 0.009002 × ICP + 0.3338, *r* = 0.8338, *P* < 0.0001, *n* = 28. CT: computer tomography; DC: decompressive craniectomy; ICP: intracranial pressure; PI: pulsatility index; RI: resistance index; SDH: subdural haematoma; TBI: traumatic brain injury

To reflect the cerebral haemodynamics of patients with malignant IOBB, we performed pre-craniotomy prospective observations by transcranial Doppler ultrasonography (Multi-Dop X1, DWL Inc., Germany) on patients with TBI who fulfilled the inclusion and exclusion criteria between January 2021 and May 2022. The waiting time for treatment of patients was shortened by reducing the ultrasonic examination time and number of body parts subjected to examination. Designated professional technicians used a 2-MHz probe to detect middle cerebral artery blood flows on the SDH-affected side via the temporal window and recorded the relevant haemodynamic parameter values. A large bone flap incision traversing the skin over Kocher’s point in the frontal area on the same side was adopted. Before bone flap creation, the frontal horn of the ventricular system was first punctured to a depth of 4–6 cm towards the sagittal plane intersection where the line connecting the bilateral external auditory canals and deformed brain midline (using the cavum septi pellucidi as reference) were located. Subsequently, an intraventricular monitoring probe (Type 82-6653, Codman, A Johnson & Johnson Inc., USA) was implanted, withdrawn from the skin via a puncture point, and connected to a cerebral function monitor for the acquisition of ICP data (Fig. 1a, b, c). This study was approved by the ethics committee of the 900th Hospital (No. 2018-005) and performed in accordance with the Declaration of Helsinki (2013 revision), all participants provided informed consent.

### Animal grouping and preparation

The animal experiment was approved by the ethics committee of Fujian Medical University (No. 2020-050) and conducted in accordance with the Guide for the Care and Use of Laboratory Animals (Institute for Laboratory Animal Research, National Research Council. Washington, DC: National Academy Press, 1996). Adult male Sprague-Dawley rats were randomly divided into four groups, namely the sham, diffuse brain injury (DBI), diffuse brain injury with concomitant 200 µL subdural haematoma (DBI + SDH200), and diffuse brain injury with concomitant 400 µL subdural haematoma (DBI + SDH400) groups. Each experimental rat was anaesthetised with 3% isoflurane gas, immobilised on a stereotactic frame, and subjected to continuous mechanical ventilation with a gas mixture containing 78% nitrogen gas, 21% oxygen gas_,_ and 1% isoflurane using a 16G polyurethane tracheal intubation catheter (Tracheal intubation suit RE20, Intelligent animal ventilator R419, RWD Life Science Co., Shenzhen, China). The body temperature of the rats was maintained at approximately 37.0 °C using heating pads. An incision was made in the right inguinal region for the insertion of a blood pressure monitoring probe into the femoral artery to obtain mean arterial pressure (MABP) and arterial blood gas (ABG) data. Using a high-speed drill (ALC-CED8, Alcott Biological Co., Shanghai, China), a 1.3-mm hole was drilled at 1.5 mm anterior to the coronal suture and at 2 mm lateral to the sagittal suture (one on each side). Cranium polishing was performed between the coronal and lambdoid sutures to create an observation zone for cerebral cortical vessels, with care taken to maintain the integrity of the cranium and to achieve good laser penetrability.

### Model construction

**Fig. 2:**
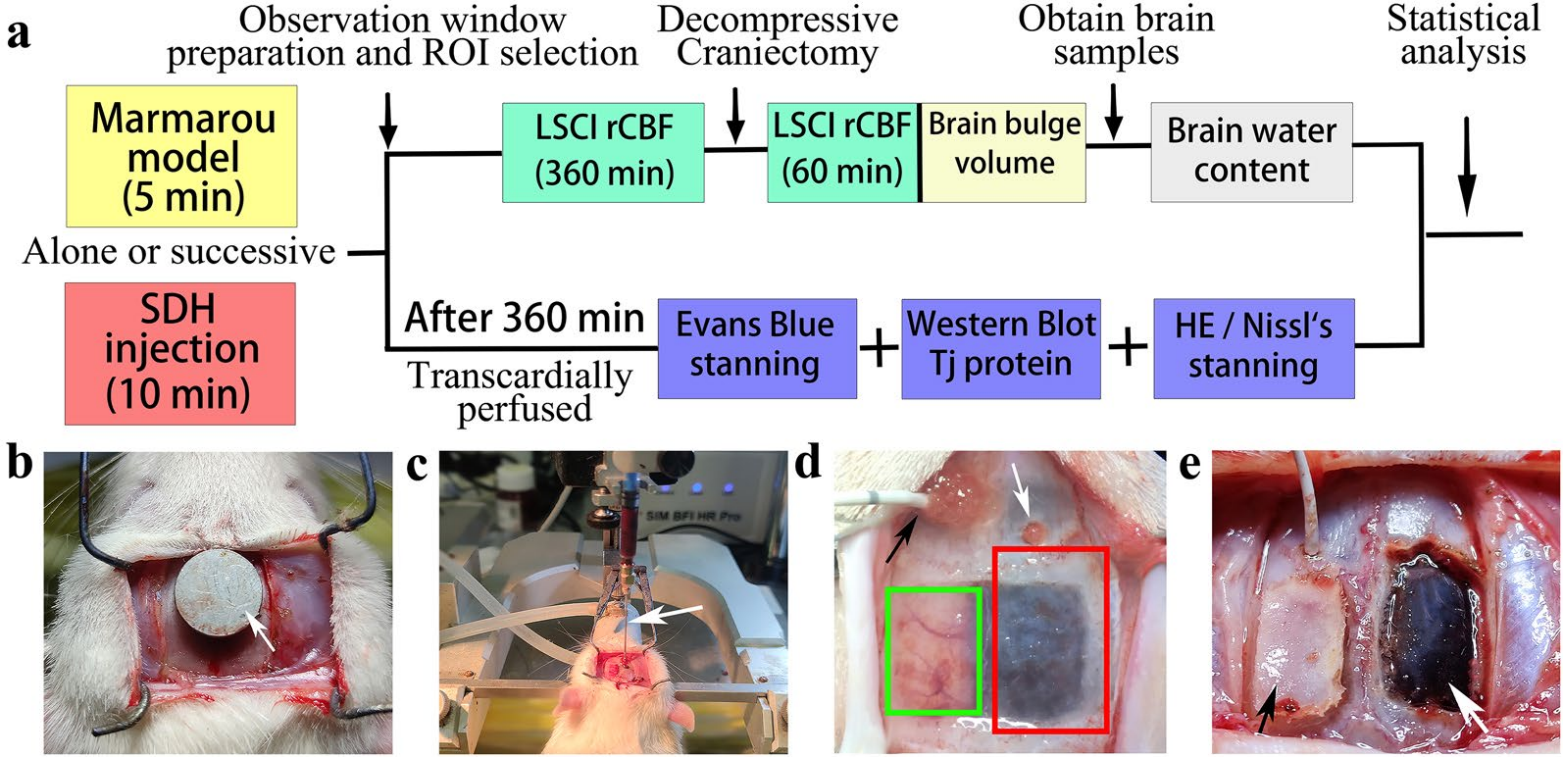
Experimental design of the rat model. **a** Experimental process. **b** In the Marmarou model, the metal disc (white arrow) was securely adhered to the cranium. **c** Using a microinjector and an 8G spindle-shaped needle (white arrow), the semi-coagulated blood mass was injected through the cranial hole on the right side. **d** Cranial holes used for ICP probe implantation (black arrow) and SDH injection (white arrow); areas shaded in red and green represent the DC bone window and LSCI observation window, respectively. **e** Bone flap removed by DC (black arrow), with the dura mater (white arrow) yet to be cut open. DC: decompressive craniectomy; ICP: intracranial pressure; LSCI: laser speckle contrasting imaging

We first constructed a rat model of DBI using the method reported by Marmarou et al. [8, 9]. A 450-g copper weight was allowed to fall freely from a height of 2 m through a glass tube onto the centre of a 10-mm metal disc attached between the coronal and lambdoid sutures of the rat, and the rat was immediately removed upon impact to prevent a second impact. Subsequently, SDH was produced by injection in accordance with the method reported by Xian et al. [10]. The dura mater was cut open at the injection site on the right side, and an 8G gavage needle with an external diameter of 1.4 mm was inserted into the cranial hole and under the dura mater. After subdural injection of a certain volume of semi-coagulated blood at a rate of 80 µL/min, the cranial hole was sealed with bone wax. Semi-coagulated blood used for haematoma production was prepared by collecting autologous fresh blood from the femoral vein and placing the collected blood in a constant-temperature water bath at 37.0 °C for 3 min to form a jelly-like semi-coagulated mass. The combined DBI-SDH model was constructed by performing SDH injection immediately after the induction of DBI had been completed. After 6 h of model observation, DC and haematoma evacuation were performed as follows: a 5 mm × 6 mm bone flap was created between the right side of the sagittal suture, coronal suture, and lambdoid suture. After bone flap removal, the dura mater was cut open for haematoma evacuation, and the cerebral cortex was exposed and subjected to further observation for 1 h. The operation procedure is shown in Fig. 2.

### Monitoring of physiological data

A catheter inserted into the right femoral artery was connected to a small animal vital sign monitor (ALC-MPA-MS, Alcott Biological Co., Shanghai, China). Subsequently, an ICP probe (Type 82-6631, Codman, A Johnson & Johnson Inc., USA) was implanted through a cranial hole on the left side, and the sensor was connected to a pressure monitor for continuous acquisition of dynamic MABP and ICP data. Blood flows of the cerebral cortical arteries and veins within the observation window were recorded using a laser speckle contrast imaging system (RFLSI III, RWD Life Science Co., Shenzhen, China). The arteries and veins in the observation zone were identified through colours, as well as branching and blood flow directions, and the vessel segments of interest were selected for the measurement of vessel diameter and blood flow values using software tools. To avoid measurement errors caused by vasodilation or vasospasms, the dynamic blood perfusion rates of the observed vessels were quantified using the formula: *Q = V *× *π *× *D*^2^/4, where *V* = blood flow velocity and *D* = vessel diameter. Lastly, the dynamic *Q* values and baseline values were normalised to obtain the relative blood perfusion rate (rBPR) values [6, 11, 12].

At 20 min after the completion of DC and haematoma evacuation, the brain bulge volume was calculated using the formula: *V* = *π *× *a *× *b *× *c *× 2/3, with *a*, *b*, and *c* being the length of bone window, width of bone window, and height of the highest point of brain tissue protrusion from the bone window plane, respectively. After all observations had been completed, rats were killed by excess halothane inhalation (5%) and brain specimens were extracted. The hemispheres were separated in the mid-sagittal plane, and the wet weight of each hemisphere was determined using an electronic balance. The hemispheres were then placed in a 95 °C oven for 5 days and separately weighed to obtain the dry weights. Brain tissue water content was calculated using the following formula: Brain tissue water content = (Wet weight − dry weight) ÷ wet weight × 100%.

### Measurement of blood–brain barrier (BBB) injury

A partial subgroup of model rats did not undergo DC at 6 h after model construction but were injected with 2% Evans blue (EB) via the femoral vein. Then, 1 h later, intracardiac perfusion was performed with heparinised saline, and the rats were decapitated for the observation of EB extravasation. Brain tissue was removed and evenly separated into the two hemispheres, which were separately weighed. Subsequently, the brain tissue was cut into small pieces, placed in dimethylformamide solution (1 mL/100 g brain tissue) for incubation at 60 °C for 24 h, centrifuged at 1000 r/min for 5 min, and subjected to absorbance measurement at 620 nm using a spectrophotometer. After the standard EB curve had been constructed, the EB content was quantified and calculated for the various brain tissues. Another portion of the model rats not subjected to DC was subjected to decapitation at 6 h after model construction for the removal of brain tissue. The contents of tight junction (TJ) proteins such as CLDN5, occludin, and ZO-1 were determined by Western blotting. Brain tissue was homogenised, loaded into an electrophoretic chamber using 10–15% sodium dodecyl sulphate–polyacrylamide gel electrophoresis gel, and transferred onto a polyvinylidene fluoride (PVDF) membrane (Immobilon-P, Millipore, Bedford, MA, USA). After blocking, the PVDF membrane was sequentially incubated with the primary antibodies (β-actin 1:1000, ZO-1 1:200, Occludi 1:1000, CLDN5 1:500) and rabbit secondary antibodies (1:5000), and band images were acquired using an antibody-conjugated gel imaging system. Greyscale values of bands were analysed using Image J, and normalisation was performed to determine the relative contents of the target proteins.

### Pathomorphological observations

A partial subgroup of rats were not subjected to DC at 6 h after model construction but received sequential intracardiac perfusions of heparinised saline and 4% paraformaldehyde solution (pH 7.4). Brain tissue was dehydrated, fixed, embedded in paraffin, and sectioned at a thickness of 3 mm along the coronal plane. Certain sections were stained with haematoxylin and eosin (HE staining), while others were stained with toluidine blue solution (Nissl's staining). The pathomorphological characteristics of the cerebral cortex of rats belonging to different groups were observed under an optical microscope.

### Statistical analyses

Statistical analyses were performed using SPSS (Version 26.0, SPSS Inc. Chicago, IL, USA), and all data were expressed as means ± standard deviations. Based on the clinical data of patients included in this study, binary classification was performed on eight suspected causes of malignant IOBB, as follows, IOBB (no 0, yes 1), sex (female 0, male 1), age (≥ 60 years 0, < 60 years 1), preoperative GCS score (> 6 points 0, ≤ 6 points 1), concomitant multiple contusions and lacerations in bilateral brain tissue (no 0, yes 1), concomitant SAH or intraventricular haemorrhage (no 0, yes 1), bilateral basal cistern compression and disappearance (no 0, yes 1), SDH thickness (< 10 mm 0, ≥ 10 mm 1), ICP value before craniotomy (< 40 mmHg 0, ≥ 40 mmHg 1). The possible risk factors were selected by univariate analysis, and high-risk factors were subsequently identified by binary logistic regression analysis (Forward, Entry 0.05, Removal 0.10). ICP values and pulsatility index (PI)/resistance index (RI) values were plotted on a scatter plot and subjected to Pearson’s linear correlation analysis. The differences in data among the rat model groups were compared by one-way analysis of variance (ANOVA) and repeated measures ANOVA, with differences considered statistically significant when *P* (two-tailed) < 0.05.

## Results

### High-risk factors for malignant brain bulge during DC

The data of 113 patients were retrospectively analysed. These patients comprised 82 men and 31 women with an average age of 50.4 ± 15.7 years and included 21 patients who developed malignant IOBBs. The results of the one-way ANOVA showed that sex and concomitant multiple contusions and lacerations in bilateral brain tissue did not show. Among the remaining independent variables that were included in the binary logistic regression model for the analysis, age, preoperative GCS score, bilateral basal cistern compression and disappearance and ICP value before craniotomy were ultimately included in the regression equation and these demonstrated statistical significance (Table 1). Therefore, patients aged < 60 years, with a preoperative GCS score ≤ 6, compression and disappearance of bilateral basal cisterns revealed by preoperative imaging, and pre-craniotomy ICP ≥ 40 mmHg were significantly associated with an elevated risk of developing malignant IOBB. Among the patients who developed IOBB, 12 had a 3-month postoperative Glasgow Outcome Scale score of 1 (seven patients died at 1 week postoperatively), five had a score of 2, and four had a score of 3. Patients with IOBB also had a significantly higher overall 3-month postoperative mortality compared with other patients (57.1% vs 10.9%, *P* < 0.001).

**Table 1:**
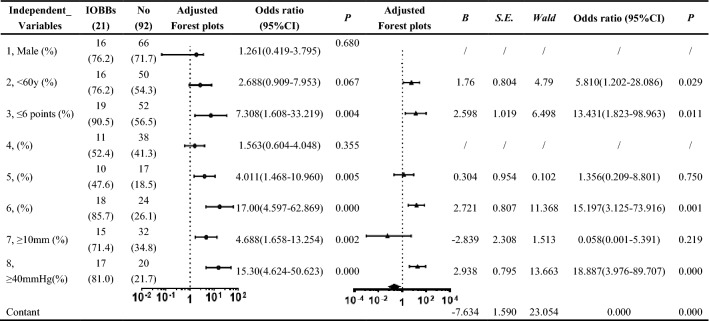
Statistical analysis of malignant IOBB during DC in patients with severe TBI and concomitant SDH

(1) Sex; (2) age; (3) preoperative GCS score; (4) concomitant multiple contusions and lacerations in bilateral brain tissue; (5) concomitant SAH or intraventricular haemorrhage; (6) bilateral basilar cistern compression and disappeared; (7) SDH thickness; (8) ICP value before DC

DC: decompressive craniectomy; GCS: Glasgow Coma Scale; ICP: intracranial pressure; IOBB: intraoperative brain bulge; SAH: subarachnoid haemorrhage; SDH: subdural haematoma; TBI: traumatic brain injury

### Middle cerebral artery blood flows of patients

A total of 28 patients were monitored during the scheduled period, of whom six developed malignant IOBB. Besides possessing all four high-risk factors as stated above, these six patients also exhibited low flow and high resistance characteristics, in the spectra of middle cerebral artery blood flow on the SDH-affected side (Fig. 1d). Tall, sharp systolic peaks and the disappearance of diastolic peaks in the spectra indicate a substantial decrease in middle cerebral artery blood flow. In addition, the PI and RI values were also significantly higher than those of patients without malignant brain bulge, with differences being statistically significant. Scatter plot analysis of the data of the 28 patients revealed the presence of significant linear correlations between the PI/RI and ICP values (Fig. 1e). The PI and RI increased with an increase in ICP, with the PI and ICP showing higher sensitivity and accuracy. The abovementioned results demonstrate that an excessive increase of ICP ≧ 40 mmHg in patients with malignant IOBB leads to high resistance to intracranial arterial blood supply and a decrease in arterial blood flow continuity, which may trigger a cascade of responses.

### Responses of rats to the single impact-acceleration factor

No deaths occurred among the rats as the experiment was performed under conditions of continuous mechanical ventilation. The body weights, as well as the baseline MABP and ICP values, of rats of the various groups before model construction were consistent. ABG indicators before model construction and after 6 h of observation were also consistent, with intra- and inter-group differences being statistically insignificant. These results demonstrate that mechanical ventilation did not cause damage to the interior milieu of the rats. Table 2 shows the comparison of the various indicators of the rat model.

**Table 2:** Comparison of the various indicators in the rat model

	Sham (*n* = 5)	DBI (*n* = 10)	DBI + SDH200 (*n* = 10)	DBI + SDH400 (*n* = 10)	*F*	*P*
Weight (g)	332.35 ± 12.85	328.19 ± 10.45	328.56 ± 15.15	327.06 ± 14.85	0.174	0.913
Normal MABP (mmHg)	87.74 ± 3.70	89.92 ± 5.15	90.45 ± 5.20	90.45 ± 3.28	0.492	0.690
Normal ICP (mmHg)	4.55 ± 0.96	4.31 ± 0.53	4.21 ± 0.56	4.54 ± 0.95	0.448	0.720
Normal ABG index						
PH	7.40 ± 0.02	7.39 ± 0.04	7.40 ± 0.02	7.40 ± 0.03	1.89	0.152
PO2	130.00 ± 5.06	130.94 ± 8.90	135.56 ± 9.44	132.20 ± 4.96	0.844	0.480
PCO2	39.18 ± 2.36	39.75 ± 2.85	38.55 ± 3.07	38.46 ± 2.40	0.474	0.702
ABG index 6 h after modelling						
PH	7.40 ± 0.07	7.36 ± 0.04	7.38 ± 0.03	7.39 ± 0.05	1.109	0.360
PO2	132.12 ± 4.56	126.59 ± 3.78	132.56 ± 8.54	128.75 ± 4.42	2.146	0.114
PCO2	36.20 ± 1.97	37.67 ± 3.52	35.88 ± 2.59	36.24 ± 2.73	0.755	0.528

ABG: arterial blood gas; DBI: diffuse brain injury; ICP: intracranial pressure; MABP: mean arterial pressure; SDH: subdural haematoma

**Fig. 3:**
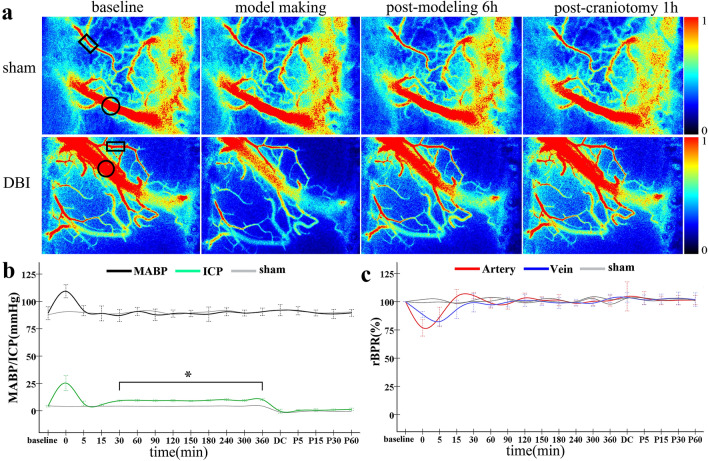
Data monitoring for rats of the DBI group. **a** LSCI monitoring of blood flow in the arteries and veins of interest in the cerebral cortex of the non-SDH affected-side before model construction, immediately after model construction, 360 min after model construction, and 60 min after DC; rectangles denote the arteries of interest, and circles denote the veins of interest. **b** Dynamic changes in the MABP and ICP values of the sham and DBI groups at various time points. **c** Dynamic changes in the rBPR of the arteries and veins of the sham and DBI groups at various time points. Scale bar = 1 mm. *n* = 5 in the sham group and 10 in the DBI group. *P < 0.05. DBI: diffuse brain injury; DC: decompressive craniectomy; ICP: intracranial pressure; LSCI: laser speckle contrast imaging; MABP: mean arterial pressure; rBPR: relative blood perfusion rate; SDH: subdural haematoma

In the impact-acceleration single factor injury induction model, the rats showed reactive increases in the MABP and ICP when subjected to injury induction. The MABP values did not exhibit significant changes after injury induction and after DC. By contrast, the ICP values increased gradually after induction and stabilised after 30 min, with the mean value being 9.6 ± 2.1 mmHg and significantly higher than that of the sham group (*P* < 0.05). The ICP values remained close to 0 after DC (Fig. 3b). The CBF values of the arteries and veins decreased initially after injury induction but subsequently rebounded, with the highest values exceeding the baseline values and the rebound being almost concurrent in the arteries and veins. After 30 min, the CBF returned to the baseline level in both the arteries and veins and it was observed that DC had no significant effects on the CBF values of the cortical arteries and veins on the contralateral side (Fig. 3c).

### Violent physiological responses in DBI rats following the addition of SDH

**Fig. 4:**
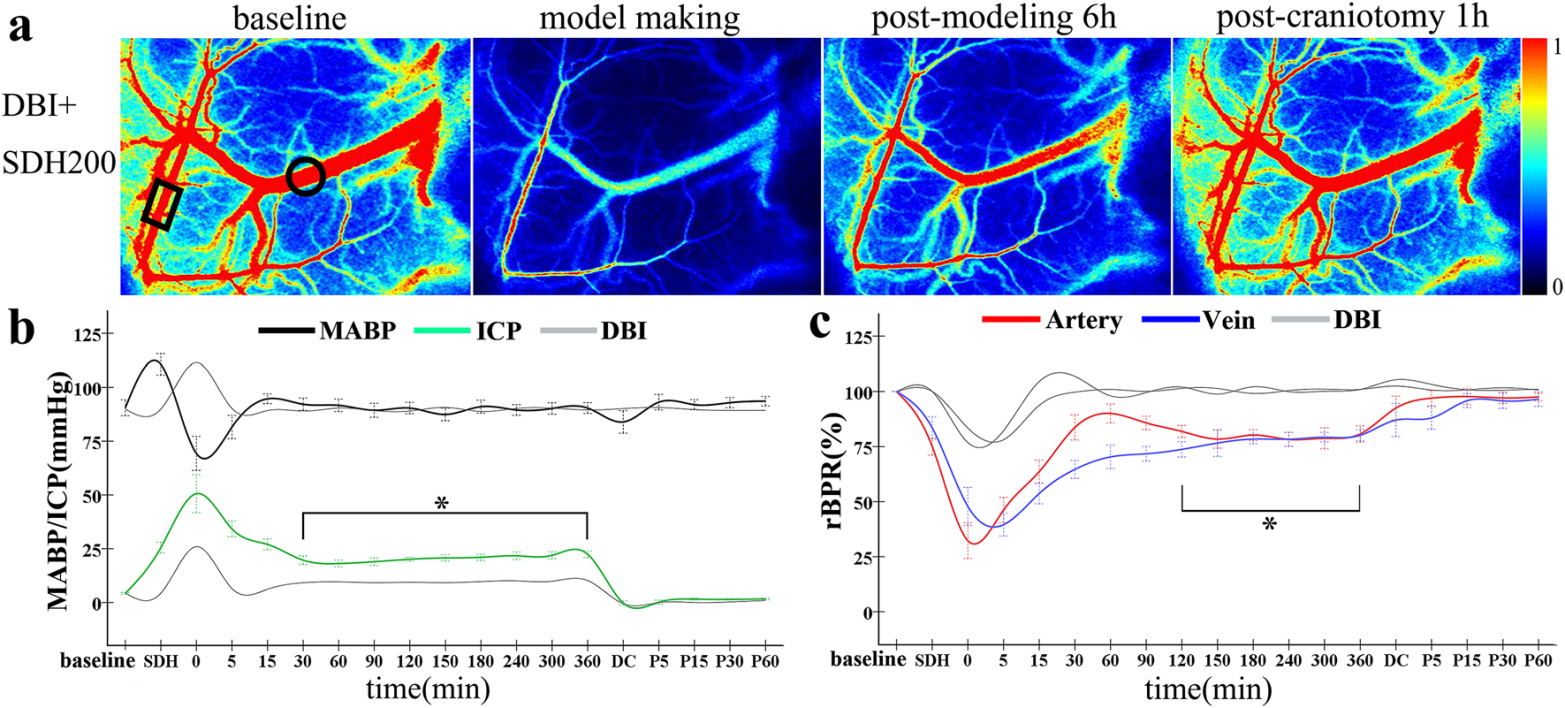
Data monitoring for rats of the DBI + SDH200 group. **a** LSCI monitoring of blood flow in the arteries and veins of interest in the cerebral cortex of the non-SDH affected-side before model construction, immediately after model construction, 360 min after model construction, and 60 min after DC; rectangles denote the arteries of interest, and circles denote the veins of interest. **b** Dynamic changes in the MABP and ICP values of the DBI + SDH200 group at various time points. **c** Dynamic changes in the rBPR of the arteries and veins of the DBI + SDH200 group at various time points. Curves of dynamic changes were compared with those of the DBI group. Scale bar = 1 mm. *n* = 10 per group. *P < 0.05. DBI: diffuse brain injury; DC: decompressive craniectomy; ICP: intracranial pressure; LSCI: laser speckle contrast imaging; MABP: mean arterial pressure; rBPR: relative blood perfusion rate; SDH: subdural haematoma

In the DBI + SDH200 group, a temporary trough occurred in the MABP after haematoma injection, followed by a return to the baseline level. The effect of DC on the MABP was not significantly different in the DBI + SDH200 group from that of the DBI group. ICP increased continuously with SDH injection and reached a maximum value of 50.6 ± 12.3 mmHg. After the injection had been completed, ICP decreased slowly within 30 min and was stabilised at 21.9 ± 3.1 mmHg between 30 and 360 min. The ICP values at the various times points were different from the corresponding values of the DBI group, with the differences being statistically significant (*P* < 0.05). The ICP values remained close to 0 after DC (Fig. 4b). The CBF values of the cortical arteries and veins in the non-SDH-affected side initially decreased rapidly after injection and subsequently increased gradually, with the rate of increase being higher in arteries than in veins. The rBPR was stabilised at the baseline level of 76–82% after 120 min, and the rBPR of the arteries and veins at the various time points in the DBI + SDH200 group were significantly different from the corresponding values of the DBI group (both *P* < 0.05). After DC, the CBF values of the arteries and veins increased to the baseline level, with the rate of increase as previously mentioned, being higher in the arteries than in the veins. The rBPR values at the various time points were not significantly different from those of the DBI group (Fig. 4c). Our results show that the SDH factor increased the ICP of DBI rats and concurrently retarded the restoration of cerebral cortical blood flow. DC led to a reduction of ICP and caused a rapid restoration of cerebral cortical blood flow, and the arteries exhibited a quicker response to sudden changes in ICP compared with the veins.

**Fig. 5:**
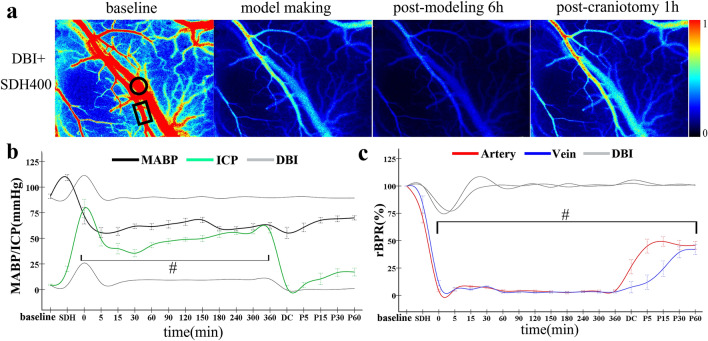
Data monitoring for rats of the DBI + SDH400 group. **a** LSCI monitoring of blood flow in the arteries and veins of interest in the cerebral cortex of the non-SDH affected-side before model construction, immediately after model construction, 360 min after model construction, and 60 min after DC; rectangles denote the arteries of interest, and circles denote the veins of interest. **b** Dynamic changes in the MABP and ICP values of the DBI + SDH400 group at various time points. **c** Dynamic changes in the rBPR of the arteries and veins of the DBI + SDH400 group at various time points. Curves of dynamic changes were compared with those of the DBI group. Scale bar = 1 mm. *n* = 10 per group. *P < 0.05, ^#^*P* < 0.01. DBI: diffuse brain injury; DC: decompressive craniectomy; ICP: intracranial pressure; LSCI: laser speckle contrast imaging; MABP: mean arterial pressure; rBPR: relative blood perfusion rate; SDH: subdural haematoma

In the DBI + SDH400 group, the MABP values decreased initially and increased subsequently following double-volume haematoma injection. The MABP did not return to baseline level within 360 min or after DC. ICP reached a peak value of 79.1 ± 12.4 mmHg during injection, decreased slowly within 30 min of injection, and subsequently increased to 56.9 ± 2.3 mmHg. The ICP values at various time points were significantly higher than the corresponding values of the DBI and DBI + SDH200 groups (both *P* < 0.01). ICP decreased rapidly after DC but increased again when the brain bulge occurred (Fig. 5b). The rBPR values of the cerebral cortical arteries and veins on the non-SDH-affected side decreased to < 10% with haematoma injection and did not recover within the 360 min observation time frame. After DC, the CBF of the arteries and veins increased gradually and the rBPR reached 41–45% of baseline level at 60 min, with the values at different time points being significantly different from the corresponding values of the DBI and DBI + SDH200 groups (both *P* < 0.01). The rate of restoration of arterial blood flow after DC was still higher than that of venous blood flow, with the latter exhibiting a lag effect (Fig. 5c). Therefore, a larger SDH further reduced the capacity of the cranial cavity, which not only caused an extreme increase in ICP and reactive decrease in the MABP, but also led to inadequate cerebral perfusion pressure, thereby resulting in interrupted CBF in the cerebral cortex, and ischaemia and hypoxia in bilateral brain tissue. Although DC enabled the expansion of cranial cavity capacity, the development of brain bulge caused another increase in ICP, resulting in the inability of the partially restored CBF to satisfy the blood flow requirement for cerebral perfusion.

### Malignant brain bulge and brain tissue water content

**Fig. 6:**
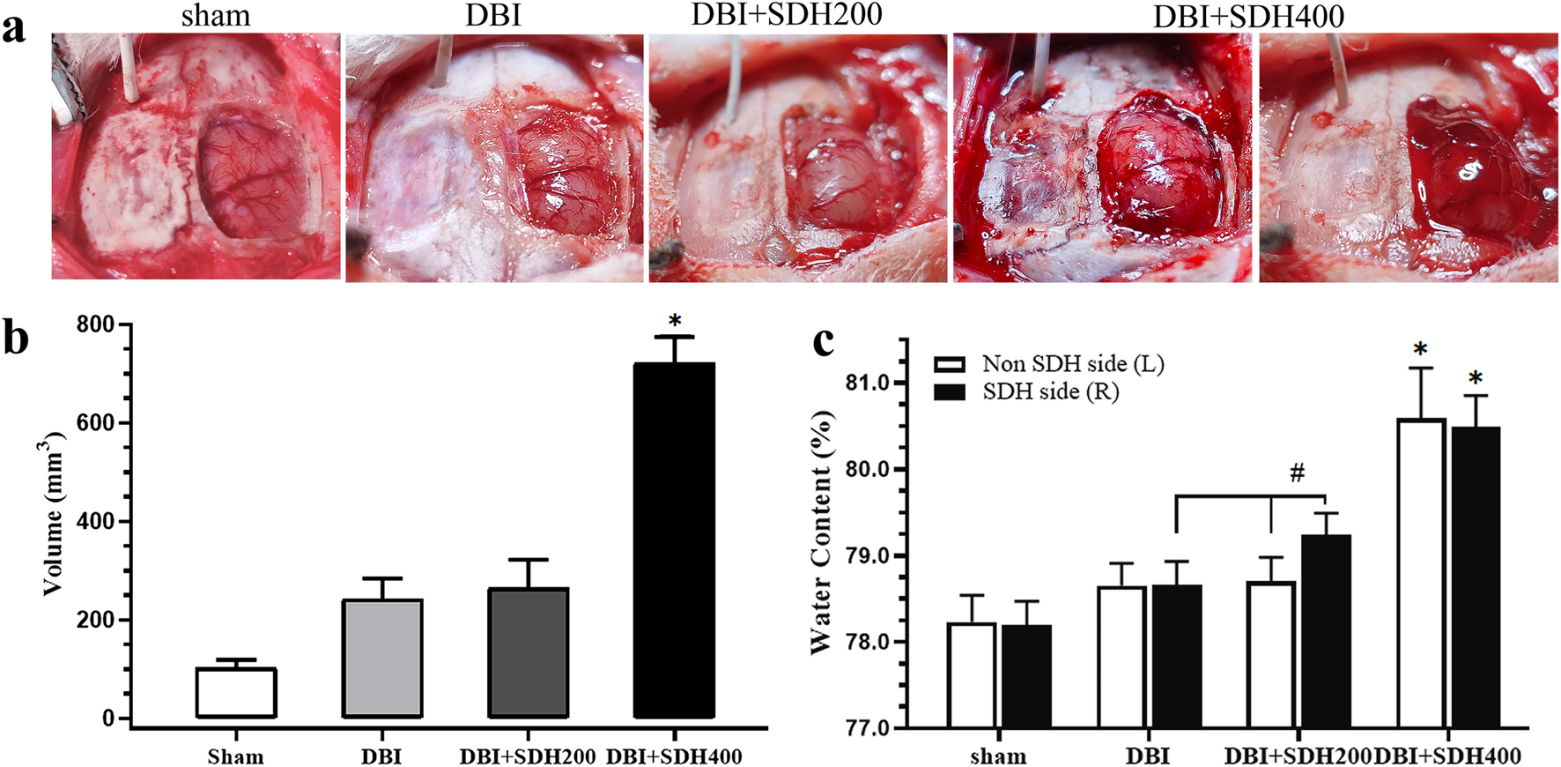
Comparison of brain bulge volume and brain water content among rats of various groups. **a** After the addition of 400 µL SDH, the brain tissue clearly bulged beyond the bone window and was accompanied by pia mater damage and haemorrhage. **b** The brain bulge volume of the DBI + SDH400 group was significantly larger than that of the other groups. **c** Comparison of brain water content revealed that the brain tissue on the SDH-compressed side had a significant increase in brain water content, and the DBI + SDH400 group also had a significant increase in brain water content on the non-compressed contralateral side. *n* = 5 in the sham group and 10 per group in the rest. *P < 0.05, ^#^*P* < 0.01. DBI: diffuse brain injury; SDH: subdural haematoma

At 20 min after the removal of the right bone flap and haematoma evacuation, slight bulges were observed in the brain tissue of the rats of the sham group, with the volume being 105.8 ± 33.5 mm^3^. Both the DBI and DBI + SDH200 groups also exhibited brain bulges with volumes of approximately 243.0 ± 57.9 mm^3^. At the 60-min observation time point, the brain bulges were exacerbated but trapping and compression of brain tissue in the bone window were not observed in the DBI and DBI + SDH200 groups. By contrast, the DBI + SDH400 group experienced significant brain tissue trapping and compression accompanied by blood oozing and damage in the pia mater at 20 min, with the volume being 722.5 ± 73.3 mm^3^ (Fig. 6a, b). The differences in brain bulge volume between the DBI + SDH400 group and the other three groups were statistically significant (both *P* < 0.01).

Water content of the bilateral brain tissue of the DBI group was increased compared with the sham group, although the water content of the left and right sides did not differ significantly. In the DBI + SDH200 group, the water content of the SDH-compressed right brain tissue was higher than that of the left-brain and bilateral brain tissues of the DBI group, with the differences being statistically significant (both *P* < 0.05). In the DBI + SDH400 group, the brain tissue of both sides showed a sharp increase in brain water content. The differences in brain water content compared with the other three groups were statistically significant (both *P* < 0.01), although the difference between the left and right brain tissues was not significant (Fig. 6c). The abovementioned results demonstrate that the SDH compression factor caused a significant increase in the brain water content of the hemisphere that received the haematoma injection. However, when double-volume SDH injection was performed, a sharp increase in brain water content also occurred in the non-SDH-affected hemisphere.

### BBB injury in rats

**Fig. 7:**
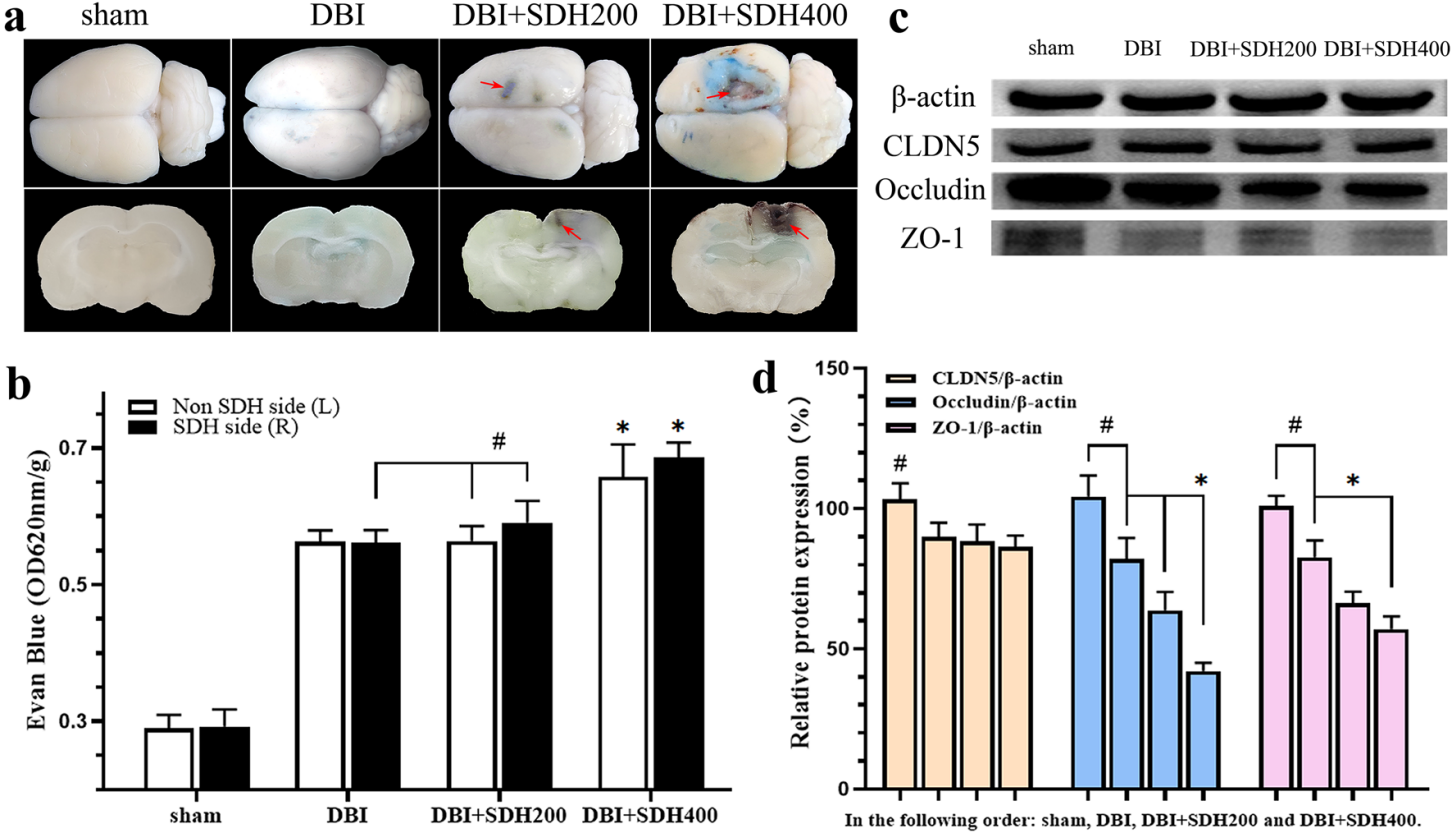
BBB and TJ protein damage in the various groups. **a** Observation of the overall EB-stained brain tissue and coronal sections shows the presence of EB extravasation accompanied by obvious localised haemorrhagic foci after compression by SDH (red arrows). **b** Comparison of the proportion of EB extravasation at an absorbance of 620 nm/g. **c** Western blot analysis of TJ protein expression in various groups. **d** Quantitative analysis of CLDN5, Occluding, and ZO-1 protein expression using the β-actin normalisation method. *n* = 5 in the sham group and 10 per group in the rest. *P < 0.05, ^#^*P* < 0.01. BBB: blood–brain barrier; CLDN5: Claudin proteins; EB: Evans blue; SDH: subdural haematoma; TJ: tight junction

The brain tissue of rats belonging to the DBI group exhibited diffuse EB extravasation. In groups with the additional SDH factor, localised clustered extravasation accompanied by bleeding in the right cortex occurred in addition to diffuse extravasation. Double-volume SDH injection aggravated the phenomenon of clustered extravasation accompanied by bleeding (Fig. 7a). The amount of EB extravasation in the bilateral brain tissue was significantly greater in the DBI group than in the sham group. Extravasation was increased in the right brain tissue of the DBI + SDH200 group compared with the left-brain tissue of the same group and bilateral brain tissue of the DBI group (Fig. 7b), while extravasation in the bilateral brain tissue of the DBI + SDH400 group was significantly increased compared with all other groups (both *P* < 0.01). Compared with the sham group, the other three groups showed significant decreases in the expression of TJ proteins, which were associated with different SDH volumes, especially in the expression of occludin and ZO-1 (Fig. 7c, d). Therefore, our results demonstrate that impact-induced DBI caused damage to the BBB of rats and that the compression exerted by SDH further exacerbated the damage, with a large volume of SDH being capable of inducing BBB damage in bilateral brain tissue.

### Pathomorphological damage in rats

**Fig.8:**
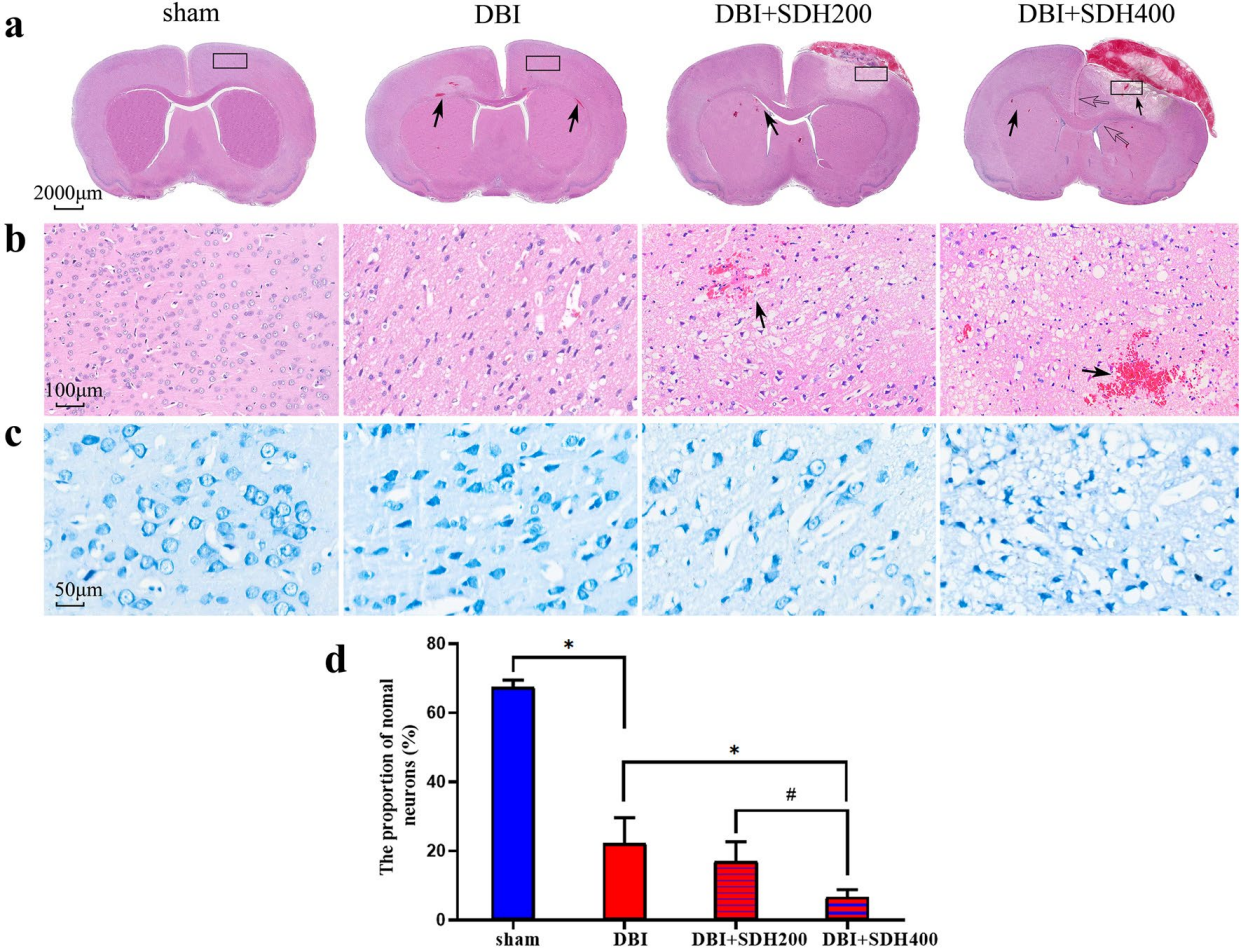
Pathological sections of paraformaldehyde-perfused brain tissue of the various groups. **a** General observation of HE-stained tissue reveals the presence of localised intraparenchymal haemorrhagic foci (black arrows) and shifting of the ventricles and cisterns (hollow arrows) under compression. **b** Observation of the same HE-stained local region in the right cerebral cortex of all groups (100um, 20 ×). **c** Observation of the same Nissl-stained local region in the right cerebral cortex of all groups (50um, 40 ×). **d** Comparison of a normal neuron count within a 50-µm field of view in Nissl-stained sections of the same region in the right cerebral cortex of the various groups. *n* = 3 in the sham group and 6 per group in the rest. *P < 0.05, ^#^*P* < 0.01. HE: haematoxylin and eosin

General observation of HE-stained sections revealed the presence of progressive damage in the brain tissue of all four groups of rats (Fig. 8a). The specimens of the DBI group exhibited intraparenchymal haemorrhagic foci caused by the impact injury and narrower ventricles and cisterns compared with the sham group. In rats with the additional SDH factor, the cerebral cortex on the SDH-affected side exhibited obvious injury with cell death and loose cytoplasm. Under the compressive effects of 400 µL SDH, the cerebral cortical damage was aggravated, with brain tissues showing obvious haemorrhagic foci, disappearance of ventricles and cisterns under compression, and a shift in midline structure. Cerebral cortical tissue from the same site on the right side of the brain was selected for the observation of HE-stained sections at 20 × magnification and Nissl-stained sections at 40 × magnification (Fig. 8b, c). The sham group had no significant pathological changes and exhibited neurons with clear contours, large round nuclei, light staining, weakly acidophilic cytoplasm, and narrow bands of brightness around the cells. Specimens of rats of the DBI group showed swelling, blurred contours, little pyknosis and unclear cytoplasmic boundaries in certain neurons, looseness in the surrounding cells, and larger intervascular spaces. With the addition of the SDH factor, the stained cerebral cortical sections compressed by SDH exhibited a worse state with generalised swelling, necrotic nuclei, blurred cytoplasmic contours, large patches of looseness among surrounding cells, and larger intervascular spaces accompanied by intraparenchymal haemorrhagic foci, these changes were more severe in rats of the DBI + SDH400 group. The normal neuron count being significantly different in the DBI group from the sham group (*P* < 0.05) and in the DBI + SDH400 group from the DBI and DBI + SDH200 groups (Fig. 8d).

## Discussion

The results of the present retrospective study indicated that patients aged < 60 years, with preoperative GCS score ≤ 6, disappearance of bilateral basal cisterns, and pre-craniotomy ICP ≥ 40 mmHg had a significantly elevated risk of developing malignant IOBB during DC. Therefore, surgeons should pay particular attention to such patients to prevent the possible occurrence of IOBB. Before surgery, transcranial Doppler ultrasonography can be adopted to determine the PI and RI values of cerebral arteries, as the presence of high-resistance and low-flow characteristics provides certain reference values for the prediction of IOBB. During the animal modelling stage of this study, we found that an increase in SDH volume concurrently increased ICP and led to decreases in CBF in the cerebral cortex to varying extents, while DC enabled the rapid relief of ICP and improvement of blood perfusion in brain tissue. However, an extreme increase in ICP led to a gradual loss of cerebrovascular function and the decrease in CBF caused severely inadequate brain tissue perfusion. Such a situation involved the uncompressed contralateral hemisphere and induced widespread damage to the BBB and neurons throughout the entire brain, which formed the basis for DBS. Although ICP could be rapidly reduced by DC, the incomplete restoration of CBF and heterogeneity in the restoration rates of cerebral arteries and veins (manifested as a significant decrease in the rate of venous return) under such circumstances, combined with DBS induced the rapid protrusion of brain tissue beyond the bone window, thereby resulting in the formation of IOBB.

The issue of brain bulge during DC, especially primary brain bulge, has rarely been investigated by researchers [13, 14]. DBS is one of the causes of primary IOBB. Severe TBI occurs as a result of the combined effects of mechanisms of injury such as acceleration/deceleration or rotation and manifests as diffuse vascular injury, diffuse axonal injury, and hypoxic-ischaemic injury. Stress-induced pathological changes arise in neurons, astrocytes, and capillaries, while BBB damage and intra- and intercellular oedema are caused by inflammation and a cascade of cell responses [15, 16]. In the animal experiment of this study, stress-induced changes occurred in the MABP, ICP, and CBF in the cerebral cortex when rats of the DBI group were subjected to impact. An acute increase in ICP led to a transient increase in the MABP and a transient decrease in the cerebral blood supply, which is akin to the Cushing response. The MABP and CBF subsequently returned to normal levels, which indicates that the cerebrovascular compensatory function remained within the normal range under certain injurious effects. However, such transient stress-induced changes still caused BBB damage and pathomorphological changes, which caused an increase in brain water content. This is consistent with previously reported results obtained with the Marmarou model [17].

Besides the primary injury, patients with severe TBI often have concomitant acute SDH as well. This is related to haemorrhage from vessels in the contused cortex and may induce generalised compression of the cerebral hemisphere [18]. When SDH was introduced into DBI rats, the MABP and ICP of the rats exhibited violent stress-induced responses, with the ICP peak value being higher under conditions of double-volume SDH. The excessively high ICP caused a gradual loss of compensation in the bodily functions of the rats and a significant decrease in the MABP and CBF in the cerebral cortex. ICP exhibited a trend of decrease after SDH injection, which was attributed to the gradual spread and dissipation of the serum separated out from the haematoma and compensatory effects of the intracranial content (Monro–Kellie curve), although the final ICP values were related to SDH volume. In the DBI + SDH200 group, the compensatory function of the cranial cavity was retained after SDH injection and reduction of ICP, although the CBF of the arteries and veins in the cerebral cortex did not return to baseline due to the space-occupying effect, and the blood flow restoration rates were also significantly lowered compared with the DBI group. Such phenomena may be more severe in the cerebral cortex of the SDH-affected side as blood flow was reduced to a greater extent near the site of compression in a rat model solely constructed by balloon compression [19]. In the DBI + SDH400 group, the body and cranial cavity of rats remained in a state of decompensation after the injection of an excessive volume of SDH. The ICP drop values still exceeded reasonable numerical interval after restoration, and the MABP did not return to the baseline level. Consequently, the closeness of the MABP and ICP values led to the non-restoration of whole-brain blood supply. The reduction or interruption of whole-brain blood supply induced ischaemia and hypoxia in brain cells, resulting in generalised BBB and neuronal injury. This agrees with the results reported by researchers in studies that used similar models [20–22]. Therefore, large-volume acute SDH and DBI exerted synergistic effects, thereby causing an increase in ICP and a decrease in the MABP. The consequent state of low perfusion, ischaemia, and hypoxia throughout the brain resulted in both vascular oedema and cellular oedema, leading to a rapid increase in brain water content and further aggravating the increase in ICP. Given the significant positive linear relationship between the ICP and RI, this resulted in another increase in resistance to CBF. Such a vicious circle caused the formation of DBS [13], which was manifested in clinical cases as a decreased GCS score and basal cistern disappearance and formed the basis for the malignant brain bulge.

At present, DC remains the primary means of treating patients with TBI when conservative therapy is incapable of attenuating an excessive increase in ICP or DBS [23]. The mechanisms of IOBB formation during DC are mixed and complex [7, 24–26]. Besides DBS, the heterogenous responses of different cerebrovascular components after decompression form another key cause of IOBB occurrence. In the DBI + SDH400 group, the CBF in the cortex was not completely restored after DC, demonstrating that the reduction of ICP is not the sole factor contributing to the improvement of CBF in the cerebral cortex. Under conditions of long-term marked increases in ICP, whole-brain perfusion was severely reduced and caused functional disturbances of the neurovascular units, as evidenced by serious BBB and histological damage. This included arterial and venous responses and the redistribution of microcirculatory blood flow [24, 27–29], which are also the initiating factors of ischaemia–reperfusion injury. However, the process of reperfusion injury is of a complex and multifactorial nature [30–32]. DC enabled a sudden decrease in ICP and induced the restoration of arterial blood supply, although the delayed response caused congestive swelling throughout the brain. Therefore, cerebrovascular autoregulation becomes impaired when major increases occur in ICP, and the delay of haematoma evacuation and decompressive surgery in clinical practice may produce irreversible injury [33].

The importance of cerebral veins in the pathophysiology of acute brain injury has attracted increasing interest from researchers in recent years [11, 19, 34, 35], and the essential effects exerted on IOBB by capillaries and the venous vasculature, which hold the greatest blood volume, cannot be overlooked. In this study, we additionally observed that the response of the veins to sudden changes in ICP was significantly delayed compared with that of the arteries. Venous walls are thinner than arterial walls and lack the characteristic smooth muscle layer of arteries. Therefore, the veins possess poorer resilience to pressure relief compared with the arteries. The elevation of ICP causes the occurrence of compression, traction, twisting, injury, and thrombosis in the superficial veins of the brain, which severely affect blood flow from the hemisphere. In addition, early blood components may also trigger a reduction in carbohydrate metabolism and an inflammatory cascade, thereby promoting hemisphere swelling or diffuse whole-brain swelling [11, 36]. Pericytes are the main mural cells for the regulation of blood flow in the capillaries. In models of ischaemic stroke [36, 37], pericytes exhibit a continuous rise in calcium ion concentration under conditions of reduced arterial supply, which can be traced to sheath-like pericytes in the arteriole-to-capillary transition zone. Such contractions are not attenuated with the restoration of arterial flow but are manifested as continuous microvascular vasoconstriction, which affects the flow of blood to the veins. The stagnation of capillary blood flow also causes neutrophil adhesion, thereby resulting in blood vessel blockage and promoting thrombosis within venules [38, 39]. Therefore, when ICP was increased in this study to the point where perfusion pressure became extremely low, the state of whole-brain ischaemia may be similar to that of ischaemic stroke, with pericytes exhibiting continuous contractions instead of recovery, following the post-DC restoration of arterial blood flow. The concurrent occurrence of thrombosis within the capillaries and venules combined with the compression, traction, and injury of the terminal veins in the cortex may also have led to a significant delay in the restoration of blood flow in the cortical veins. However, further experimental work will be required to validate these deductions, this is also the future work to be done in this study.

Acute IOBB is a critical issue that cannot be avoided during the treatment of patients with severe TBI. The occurrence of brain bulge is a dynamic process in many cases of deceleration injury-induced acute SDH. In other words, the cerebral surface has collapsed and brain pulsations are weak within the first few or up to a dozen minutes after haematoma evacuation and decompression. This is followed by the occurrence of bulging in a foam-like manner, which is caused by delayed recurrent remote site haemorrhage in certain cases. The incidence of such secondary brain bulges can be reduced through the adoption of graded or controlled decompression regimens [40, 41]. In most cases, brain bulge is manifested as mild brain tissue protrusion after decompression followed by continued protrusion beyond the bone window and consequent trapping and compression within a short period of time. Such a phenomenon is especially prevalent in patients with brain herniation before surgery and the presence of unclear basal and ambient cisterns in preoperative images. This indicates that DBS had already occurred in the patients before DC. However, DBS in humans may not be directly related to haematoma thickness and volume, as demonstrated by the non-inclusion of these factors in the regression formula in this study. A possible reason is that DBS is not merely caused by simple mechanical compression and high ICP but is also related to the degree of primary brain injury and concomitant hypoxaemia [26, 42]. DBS causes an increase in brain water content. It is not possible for brain water content to increase several-fold during the short duration of DC and to cause the rapid bulging of brain tissue. Therefore, it is certain that the process of brain bulge involves the shifting of brain contents. When DBS occurs, cerebrospinal fluid is drained from the cranial cavity and the entry of blood into the cranial cavity is hindered. During DC, the hindered arterial blood immediately rushes into the cranial cavity, although the restoration of compatible drainage rates in the capillaries and veins cannot be achieved in a timely manner. Such heterogeneity in the responses of different vasculature components forms a major cause of acute IOBB, and further studies at the capillary and venous levels are required to address the issue of CBF redistribution [20, 30, 43].

## Conclusion

In this study, an animal model of DBS was constructed by combining the Marmarou model with SDH injection to simulate the formation of malignant IOBB during DC. Subsequently, the pathological mechanisms of IOBB were explored in depth by reproducing the responses of the cerebral arteries and veins during the various stages. The utilisation of SDH volume is by no means the only method for the reduction of CBF to trigger diffuse brain swelling. Other researchers have demonstrated that DBS can also be triggered under hypoxic conditions [44]. Given the non-quantifiable nature and strong compensatory ability of the cerebrovascular system of the rat model of brain injury, the formation of DBS in the rat model differs from that in humans to a certain extent. Therefore, the results of this study can only serve as reference for clinicians. It is hoped that improved models and research protocols can be developed in the future to enable the in-depth exploration and resolution of CBF redistribution in acute IOBB. If the results of the rat experiment are to be applied to the treatment of human patients, it is recommended that clinicians identify high-risk factors for IOBB and pay particular attention to the redistribution of CBF to various vessels besides focusing on the craniotomy approach, so as to reduce the mortality and disability rates of patients.

## Data Availability

The dataset supporting the conclusions of this article is included within the article.

## Abbreviations

ABG: Arterial blood gas
ANOVA: Analysis of variance
BBB: Blood–brain barrier
CBF: Cerebral blood flow
DBI: Diffuse brain injury
DBS: Diffuse brain swelling
DC: Decompressive craniectomy
EB: Evans blue
GCS: Glasgow Coma Scale
ICP: Intracranial pressure
IOBB: Intraoperative brain bulge
MABP: Mean arterial pressure
PI: Pulsatility index
PVDF: Polyvinylidene fluoride
rBPR: Relative blood perfusion rate
RI: Resistance index
SDH: Subdural haematoma
TBI: Traumatic brain injury
TJ: Tight junction
